# Testing the causal relationships of physical activity and sedentary behaviour with mental health and substance use disorders: a Mendelian randomisation study

**DOI:** 10.1038/s41380-023-02133-9

**Published:** 2023-07-21

**Authors:** Eleonora Iob, Jean-Baptiste Pingault, Marcus R. Munafò, Brendon Stubbs, Mark S. Gilthorpe, Adam X. Maihofer, Psychiatric Genomics Consortium Posttraumatic Stress Disorder Working Group, Andrea Danese

**Affiliations:** 1 Social, Genetic & Developmental Psychiatry (SGDP) Centre, Institute of Psychiatry, Psychology & Neuroscience, King’s College London, London, UK; 2 Department of Epidemiology and Public Health, Institute of Epidemiology and Public Health, University College London, London, UK; 3 Department of Clinical, Educational, and Health Psychology, Division of Psychology & Language Sciences, University College London, London, UK; 4 Medical Research Council (MRC) Integrative Epidemiology Unit, University of Bristol, Bristol, UK; 5 School of Psychological Science, University of Bristol, Bristol, UK; 6 Physiotherapy Department, South London and Maudsley NHS Foundation Trust, London, UK; 7 Health Service and Population Research Department, Institute of Psychiatry, Psychology and Neuroscience, King’s College London, London, UK; 8 Leeds Institute for Data Analytics, University of Leeds, Leeds, UK; 9 Obesity Institute, Leeds Beckett University, Leeds, UK; 10 Alan Turing Institute, British Library, London, UK; 11 Department of Psychiatry, University of California San Diego, San Diego, CA USA; 12 Department of Child and Adolescent Psychiatry, Institute of Psychiatry, Psychology & Neuroscience, King’s College London, London, UK; 13 National and Specialist CAMHS Clinic for Trauma, Anxiety, and Depression, South London and Maudsley NHS Foundation Trust, London, UK

**Keywords:** Depression, Schizophrenia, Addiction

## Abstract

Observational studies suggest that physical activity can reduce the risk of mental health and substance use disorders. However, it is unclear whether this relationship is causal or explained by confounding bias (e.g., common underlying causes or reverse causality). We investigated the bidirectional causal relationship of physical activity (PA) and sedentary behaviour (SB) with ten mental health and substance use disorders, applying two-sample Mendelian Randomisation (MR). Genetic instruments for the exposures and outcomes were derived from the largest available, non-overlapping genome-wide association studies (GWAS). Summary-level data for objectively assessed PA (accelerometer-based average activity, moderate activity, and walking) and SB and self-reported moderate-to-vigorous PA were obtained from the UK Biobank. Data for mental health/substance use disorders were obtained from the Psychiatric Genomics Consortium and the GWAS and Sequencing Consortium of Alcohol and Nicotine Use. MR estimates were combined using inverse variance weighted meta-analysis (IVW). Sensitivity analyses were conducted to assess the robustness of the results. Accelerometer-based average PA was associated with a lower risk of depression (b = −0.043, 95% CI: −0.071 to −0.016, effect size[OR] = 0.957) and cigarette smoking (b = −0.026; 95% CI: −0.035 to −0.017, effect size[β] = −0.022). Accelerometer-based SB decreased the risk of anorexia (b = −0.341, 95% CI: −0.530 to −0.152, effect size[OR] = 0.711) and schizophrenia (b = −0.230; 95% CI: −0.285 to −0.175, effect size[OR] = 0.795). However, we found evidence of reverse causality in the relationship between SB and schizophrenia. Further, PTSD, bipolar disorder, anorexia, and ADHD were all associated with increased PA. This study provides evidence consistent with a causal protective effect of objectively assessed but not self-reported PA on reduced depression and cigarette smoking. Objectively assessed SB had a protective relationship with anorexia. Enhancing PA may be an effective intervention strategy to reduce depressive symptoms and addictive behaviours, while promoting sedentary or light physical activities may help to reduce the risk of anorexia in at-risk individuals.

## Introduction

Mental health and substance use disorders affect around one in three people across the lifespan [1] and are leading causes of the global burden of disease and disability [2, 3]. Rates of common mental disorders, such as depression and anxiety, are increasing among children and young people [4], indicating little improvement in the efficacy or implementation of current preventive strategies. Furthermore, despite several advances in psychological and pharmacological interventions, many individuals do not respond well to standard treatments [5], which also do not address the recognised physical burden of mental illness [6]. Hence, novel approaches are necessary in order to prevent and treat psychiatric disorders [7].

A growing body of evidence suggests that enhancing physical activity levels may be an effective strategy to prevent and treat mental health and substance use disorders [7, 8]. Meta-analyses examining the prospective relationship of physical activity with mental health in the population have found that higher levels of physical activity may offer protection against the onset of depression [9], stress-related disorders [10, 11], and psychotic disorders [12]. Correspondingly, prospective studies have also shown that high levels of sedentary behaviour are associated with an increased risk of these disorders [13–15]. Furthermore, meta-analyses of randomised controlled trials (RCTs) have provided evidence of the efficacy of physical activity interventions to reduce mental health symptoms and improve neurocognitive outcomes among individuals affected by depression, stress-related disorders, and schizophrenia [16]. Beyond mental health outcomes, research has also highlighted the potential beneficial role of physical activity in preventing and reducing substance use problems [17]. Observational studies suggest that physical inactivity and sedentary behaviour are linked to an increased risk of alcohol consumption and cigarette smoking [18–20]. Additionally, meta-analyses of clinical studies have found that physical exercise can effectively increase abstinence rates, reduce craving and withdrawal symptoms, and ameliorate psychological wellbeing and quality of life in people with substance use disorders [21, 22].

Despite this evidence, it is unclear whether physical activity is causally related to the risk of mental health and substance use disorders, or whether this relationship might be better explained by reverse causation and/or common causes. Although RCTs are considered the gold standard approach for establishing causality, these studies have predominantly tested the remedial effects of physical activity in at-risk samples, rather than testing its real-world protective effects in the general population. In contrast, observational prospective studies are ideally suited for studying the real-world protective effects of physical activity on psychiatric disorders. However, due to the lack of randomisation, a variety of social, behavioural, and genetic factors could be associated with both physical activity and mental health, thereby potentially acting as confounders of their relationship. Furthermore, research suggests that the relationship between physical activity and psychiatric disorders could have a bidirectional nature [23]. There is also limited evidence from well-designed prospective studies or RCTs regarding the relationship of physical activity with bipolar disorder and developmental disorders, such as attention deficit hyperactivity disorder (ADHD), autism, and eating disorders. Lastly, it is unclear whether the measurement (i.e., self-reported vs objectively assessed) and intensity of physical activity may also play a role. Research to date has predominantly used self-reported measures of physical activity, which might not accurately capture specific levels of intensity and are particularly prone to confounding by cognitive function, mood, and social desirability biases [24].

Over the past few decades, methods that exploit genetic information have been developed to overcome the limitations of RCTs and account for genetic and environmental confounding in observational studies. Mendelian randomisation (MR) is one of such methods, which uses genetic variants associated with an exposure as instrumental variables for investigating causal relationships with the outcome and vice versa [25]. This approach can reduce confounding effects since genetic variants are thought to be randomly distributed at conception, do not change over time, and cannot be affected by disease status. Earlier MR studies have found evidence of a causal protective relationship between lifestyle factors (i.e., physical activity, sleep, and diet) and psychiatric disorders [7]. With regard to physical activity, Choi et al. (2019) conducted a bidirectional MR analysis showing that accelerometer-based physical activity but not self-reported physical activity decreased the risk of depression, whereas depression was not associated with physical activity [26]. Subsequently, Sun et al. (2020) investigated the relationship of accelerometer-based overall, moderate, and sedentary activity with bipolar disorder and schizophrenia [27]. The results revealed that overall physical activity (but not moderate activity or low sedentary behaviour) was protective for bipolar disorder. In contrast, weak evidence was found for the relationship between all types of physical activity and schizophrenia. However, no study to date has used MR to test the bidirectional relationship of self-reported and accelerometer-based physical activity and sedentary behaviour with other mental health and substance use disorders (e.g., anorexia, neurodevelopmental disorders, smoking).

The present study aimed to (i) investigate the causal nature of the relationship of physical activity and sedentary behaviour with ten mental health and substance use disorders, and (ii) shed light on the causal direction of this relationship. We applied two-sample MR in order to test bidirectional associations of physical activity and sedentary behaviour with mental health and substance use disorders based on results from large genome-wide association studies (GWASs), using both self-reported and objective accelerometer-based physical activity data. An outcome-wide approach was used considering all psychiatric disorders that have been previously associated with physical activity and for which a sufficiently powered GWAS was available. Of note, both disorders previously investigated in other MR studies (e.g., depression) and novel mental health outcomes (e.g., anorexia) were included, as well as multiple physical activity exposures to assess not only the impact of the measurement method but also different levels of intensity of physical activity.

## Materials and methods

### Study design

**Fig. 1:**
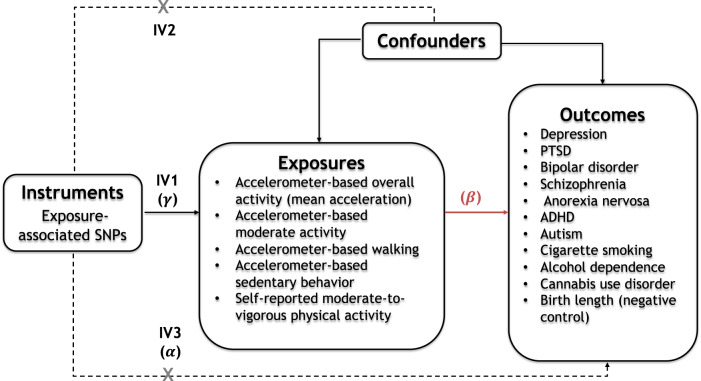
Study design and Mendelian Randomisation (MR) assumptions. Study design: Solid paths are hypothesised to exist, whereas dotted paths are hypothesised not to exist according to MR assumptions; *β* is the causal relationship of interest to be estimated, where *β* = *α/γ*. *γ* and *α* are the estimated direct effects of a SNP on the exposure and the outcome, respectively. MR assumptions: MR relies on three core assumptions for valid instrumental variables. These include: Relevance (IV1) – the instrument is associated with the risk factor of interest; Exchangeability (IV2) – the instrument is not associated with any potentially confounding variable; and Exclusion Restriction (IV3) – the instrumental variable can only influence the outcome via the risk factor (Fig. 1). In light of the first assumption, the genetic instruments were constructed using top SNPs associated with the exposure variables. The second and third assumptions are violated if instrument SNPs show horizontal pleiotropy, influencing the outcome through other causal pathways than the exposure, or correlated pleiotropy, where genetic variants for the exposure are also associated with a confounder. Therefore, several sensitivity analyses were conducted to detect and remove possible pleiotropic genetic variants, as detailed in the Methods and Results. SNP single nucleotide polymorphism.

A two-sample MR design was used to test bidirectional pathways between physical activity and mental health and substance use disorders. The analyses were conducted with physical activity as (i) the exposure, to assess whether it has a causal effect on mental health/substance use disorders, and as (ii) the outcome, to assess whether mental health/substance use disorders have a causal effect on physical activity. Summary-level data for all exposure and outcome variables were derived from large-scale, non-overlapping GWASs in individuals of European ancestry. We considered five different physical activity exposures in order to evaluate the role of different assessment methods and intensity levels. These included self-reported moderate-to-vigorous activity and accelerometer-based average activity (i.e., mean acceleration), moderate activity, walking, and sedentary behaviour. An outcome-wide approach was adopted in order to assess the causal relationship of physical activity with ten psychiatric disorders, including depression, post-traumatic stress disorder (PTSD), bipolar disorder, schizophrenia, anorexia nervosa, ADHD, autism, alcohol dependence, cannabis use disorder, and cigarette smoking. We focused on disorders for which a sufficiently powered GWAS was available (i.e., GWAS with at least one genome-wide significant locus, SNP-based heritability ≥ 0.05, and *Z*-value ≥ 4 [28]) to minimise the risk of false negative results (see Appendix 1, eMethods, eTable 1 for a description of the power of each GWAS dataset). Further, birth length was included in the analysis as a negative control outcome, as it is impossible that physical activity levels affect perinatal outcomes. Figure 1 provides an overview of the study design and the core MR assumptions for valid instrumental variables.

### GWAS data sources

(i) Physical activity and sedentary behaviour
  Summary statistics for self-reported moderate-to-vigorous activity (*N* ~ 377,000) and accelerometer-based average activity, moderate activity, walking, and sedentary behaviour (*N* ~ 91,000) were obtained from the UK Biobank [29, 30]. Self-reported moderate-to-vigorous activity during work and leisure time was calculated as the sum of total minutes per week of moderate activity (e.g., carrying light loads, cycling at normal pace) multiplied by four and the total minutes per week of vigorous activity (e.g., fast cycling, aerobics, heavy lifting) multiplied by eight in order to reflect their metabolic equivalents [29]. To objectively assess physical activity, UK Biobank participants were invited to wear a wrist-worn accelerometer at all times for 7 days. Levels of activity were measured in milli-gravity units (mg). The accelerometer data were then used to derive different phenotypes representing average activity, moderate activity, walking, and sedentary activity, which were defined using machine learning algorithms [30].
(ii) Mental health and substance use disorders

Summary statistics for diagnoses of major depressive disorder [31] (*N* ~ 143,000), PTSD (*N* ~ 956,000) (Freeze 3, Nievergelt et al., in prep.), bipolar disorder [32] (*N* ~ 413,000), schizophrenia [33] (*N* ~ 306,000), anorexia nervosa [34] (*N* ~ 69,000), ADHD [35] (*N* ~ 55,000), autism [36] (*N* ~ 46,000), alcohol dependence [37] (*N* ~ 47,000), and cannabis use disorder [38] (*N* ~ 374,000) were obtained from the Psychiatric Genomics Consortium (PGC). Summary statistics for cigarette smoking [39] (i.e., number of cigarettes smoked per day) (*N* ~ 143,000) were obtained from the GWAS and Sequencing Consortium of Alcohol and Nicotine Use (GSCAN), and those for birth length [40] (i.e., sex- and age-adjusted standardised scores) (*N* ~ 28,000) from the Early Growth Consortium (EGC). We used meta-analytic results that left out UK Biobank participants for depression, PTSD, bipolar disorder, anorexia nervosa, and cigarette smoking in order to avoid sample overlap between the exposure and outcome data. For depression, we also excluded participants from 23andMe owing to access constraints.

Further information regarding the data sources, sample size, and instrument strength of the included GWAS datasets can be found in Appendix 1 (eMethods, eTable 1). All original studies included in the GWAS datasets have been granted ethical approval, and informed consent was obtained from all participants.

### Selection of genetic instruments

We created two sets of genetic instruments for each exposure variable; the first set (G1) included only SNPs reported as genome-wide significant (*p* < 5 × 10^−8^), and the second set (G2) included top SNPs meeting a more relaxed threshold (*p* < 1 × 10^−6^). This approach of relaxing the genome-wide significance threshold for genetic instruments has been previously used in psychiatric MR research [26]. SNPs that were correlated at r^2^ > 0.001 were clumped to ensure independence between the genetic variants included as instruments. SNPs for the exposure that were not available in the summary statistics of the outcome were replaced with overlapping proxy SNPs in high-linkage disequilibrium (r^2^ > 0.8). The resulting list of SNPs used as instruments for each phenotype is shown in Appendix 2 (eTable 2).

### Statistical analyses

We considered physical activity/sedentary behaviour and mental health/substance use disorders as exposures in turn to assess potential bidirectional pathways between these. As the primary analysis, we used random-effects inverse-variance weighted (IVW) regression to combine effect estimates (i.e., Wald ratios) from multiple SNPs. For genetic instruments involving a single SNP, individual Wald ratios (WR) are presented instead. As measures of effect size, odds ratios (OR) are reported for binary outcomes and standardised beta coefficients (*β*) [41] for continuous outcomes. Given the large number of tests performed, we calculated false discovery rate (FDR) corrected p-values to account for the multiple exposures and outcomes used to test each direction of causation (55 tests in total). In sensitivity analyses, a variety of robust MR methods were used to identify and correct for potential violations of key MR assumptions, including MR-Egger, weighted median, weighted mode, MR-PRESSO, MR-RAPS, and Steiger directionality test and filtering. Additionally, we conducted Cochran’s (IVW) and Rucker’s (MR Egger) Q tests to detect heterogeneous causal effects when using meta-analytic methods. An overview of the MR methods and the rationale for their application in our study is provided in Table 1. All statistical analyses were conducted in R (version 4.0.2) using the *TwoSampleMR* package [42]. The study protocol was pre-registered in the Open Science Framework (OSF) (https://osf.io/ceptf), and any deviations that have occurred from our preregistered plans are outlined in Appendix 1 (eMethods).

**Table 1:** Description of the MR methods used in the main and sensitivity analyses.

MR method	General description	Rationale for application	Assumptions/limitations
Main analyses			
Wald ratio	• Ratio of the effect of the SNP-outcome association by the SNP-exposure association.	• Main MR method used for genetic instruments including a single SNP.	• Provides valid estimates if the genetic instrument satisfies all IV assumptions.
Inverse-variance weighted (IVW) regression [61]	• Linear regression of the SNP-outcome associations on the SNP-exposure associations, weighted by the inverse-variance of the SNP-outcome associations and with intercept constrained to zero.	• Main MR method used to combine effect estimates for genetic instruments including ≥ 2 SNPs.	• Provides valid estimates if the genetic instrument satisfies all IV assumptions. • Accounts for balanced pleiotropy (i.e., average pleiotropic effect equals to zero), but susceptible to unbalanced pleiotropy (i.e., average pleiotropic effect is positive or negative).
Sensitivity analyses			
MR-Egger regression [62]	• Weighted linear regression similar to IVW, but with intercept unconstrained.	• Provides an estimate of unbalanced horizontal pleiotropy and can yield accurate MR estimates even if all instruments are invalid. • The intercept represents the average unbalanced horizontal pleiotropic effect across SNPs.	• Makes IV1, IV2, and InSIDE assumptions (i.e., the SNP-exposure associations are independent of the direct effects of the genetic variants on the outcome). • Relaxes IV3 assumption. • But suffers from low power and is sensitive to outliers.
Weighted median method [63]	• Weighted median estimator for combining effect estimates from multiple genetic variants (instead of weighted mean as in IVW).	• The median of effect estimates is more robust to outliers than the corresponding mean (pleiotropy often manifests in the presence of genetic variants with outlying effect estimates). • Provides accurate MR estimates when the majority of the information (>50%) comes from valid instruments.	• Makes IV1 and IV2 assumptions. • Relaxes IV3 assumption.
Weighted mode method [64]	• Weighted mode estimator for combining effect estimates from multiple genetic variants (instead of weighted mean as in IVW).	• Like the median, the mode is more robust to outliers than the corresponding mean. • Provides accurate MR estimates if the largest subset of SNPs with a similar effect ratio (i.e., mode) is formed by valid instruments, even if the majority of SNPs are invalid.	• Makes IV1 and IV2 assumptions. • Relaxes IV3 assumption.
MR-PRESSO [65]	• Performs 3 tests: (1) detection of horizontal pleiotropy (global test); (2) correction for horizontal pleiotropy by removal of outliers (outlier test); (3) test for significant differences in the MR estimates before and after outlier removal (distortion test).	• Identifies and removes horizontal pleiotropic outliers in instruments including multiple SNPs.	• Makes IV1 and IV2 assumptions. • Relaxes IV3 assumption. • Best suited when horizontal pleiotropy occurs in < 50% of instruments.
Robust adjusted profile score (MR-RAPS) [66]	• SNPs are assigned different weights according to the strength of their associations.	• Allows for the use of weaker instruments, which is not recommended for other methods. • In our study, MR-RAPS is only used for the G2 instruments, which have been constructed using a more liberal *p*-value threshold and are therefore more susceptible to weak instrument bias.	• Makes IV2 and IV3 assumptions. • Relaxes IV1 assumption.
Steiger directionality test and filtering [67]	• Steiger Z-test assesses whether the absolute correlation of the genetic variants with the exposure is larger than that with the outcome. • If *Z*-value > 0, X causes Y; if *Z*-value < 0, Y causes X; if *Z* = 0, neither direction is accepted. • Steiger filtering can then be used to correct for potential misspecification of the direction of effect by removing genetic variants that explain more variation in the outcome than the exposure.	• Indicates the direction of the causal association (sign of *Z*-value) and the confidence level of the direction (*p*-value). • Identifies and removes genetic variants whose direction of effect has been misspecified.	• Results may be biased in the presence of horizontal pleiotropy or different levels of measurement error between the exposure and the outcome.

*MR* Mendelian randomisation, *SNP* single nucleotide polymorphism, *IV* instrumental variable.

## Results

**Fig. 2:**
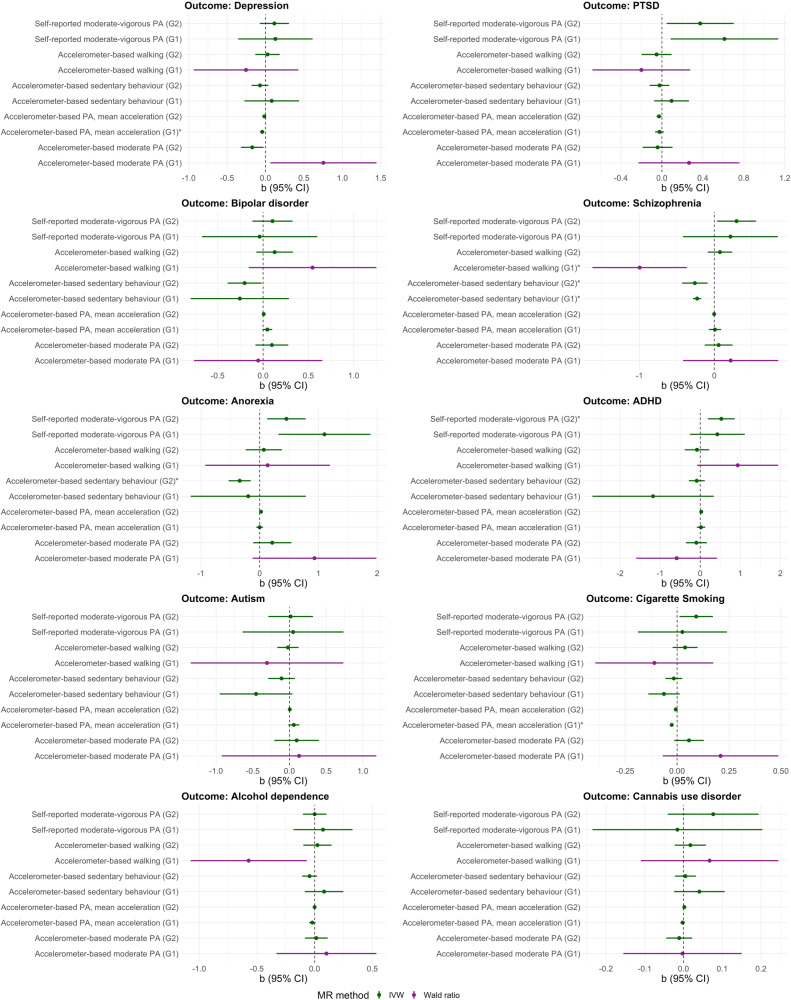
MR estimates (IVW/Wald ratio) and 95% confidence intervals for the causal relationships of physical activity and sedentary behaviour with mental health and substance use disorders (Direction 1). MR Mendelian randomisation, IVW inverse variance weighted, G1 = genome-wide significant genetic instrument (*P* < 5 × 10^−8^), G2 = more relaxed genetic instrument (*P* < 1 × 10^−6^); PA physical activity. IVW is used for analyses involving ≥ 2 SNPs, and Wald ratio for analyses involving 1 SNP. Effects marked with an asterisk (*) are robust to the correction for multiple testing (i.e., FDR-adjusted *p* < 0.05).

**Fig. 3:**
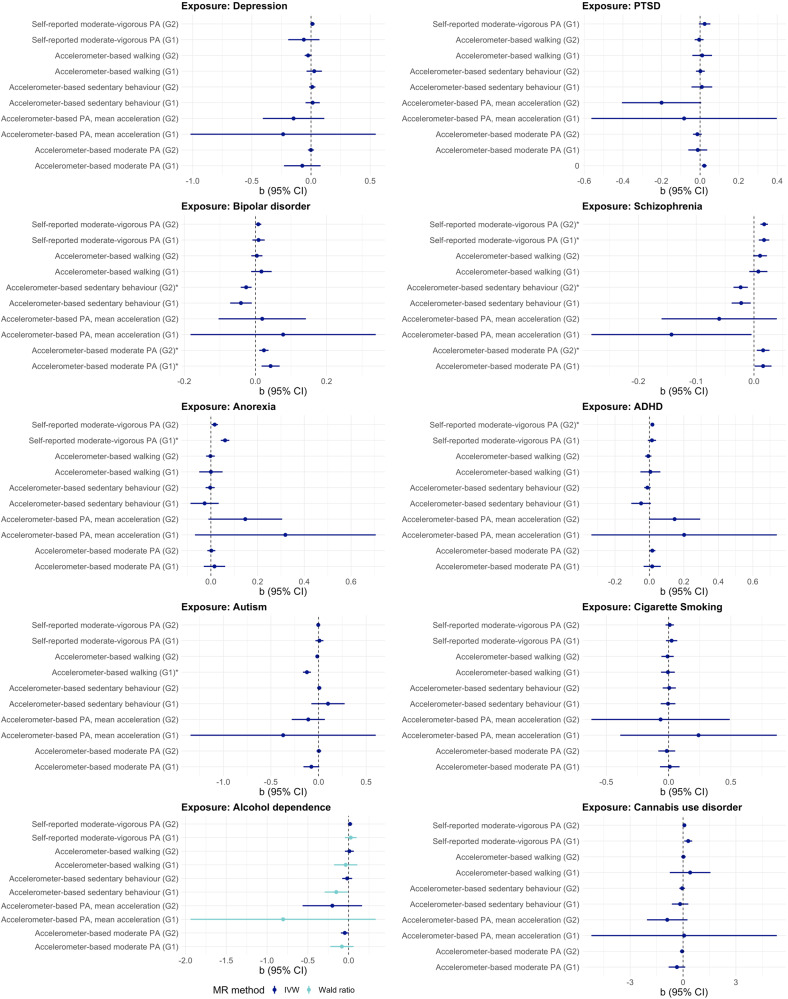
MR estimates (IVW/Wald ratio) and 95% confidence intervals for the causal relationships of mental health and substance use disorders with physical activity and sedentary behaviour (Direction 2). MR Mendelian randomisation, IVW inverse variance weighted, G1 genome-wide significant genetic instrument (*P* < 5 × 10^−8^); G2 = more relaxed genetic instrument (*P* < 1 × 10^−6^), PA physical activity. IVW is used for analyses involving ≥2 SNPs, and Wald ratio for analyses involving 1 SNP. Effects marked with an asterisk (*) are robust to the correction for multiple testing (i.e., FDR-adjusted *p* < 0.05).

The results of the main MR analyses (IVW/WR) are illustrated in Figs. 2, 3. The sensitivity analyses with MR-Egger, weighted median, weighted mode, and MR-RAPS are shown in eFigures 1, 2 (Appendix 1) and are also reported in eTables 3–6 (Appendix 2). The results of other sensitivity analyses, including MR-Egger intercept, Q statistics, MR-PRESSO, and Steiger directionality test/filtering, are shown in eTable 2 and eTables 7–11 (Appendix 2). In the following sections, we focus on the results that were robust to the correction for multiple testing (i.e., FDR-adjusted *p* < 0.05), which are also reported in Table 2.

**Table 2:** Main MR results and sensitivity analyses for the bidirectional relationships of physical activity and sedentary behaviour with mental health and substance use disorders.

Direction 1: Physical activity/sedentary behaviour -> Mental health/substance use disorders										
Outcome	Exposure	MR method	N (snps)	b	SE	CI (lower)	CI (upper)	*P*-value (raw)	*P*-value (FDR-adjusted)	Odds ratio^a^/Standardised effect^b^
Depression	Accelerometer-based PA, mean acceleration (G1)	IVW	7	**−0.043**	**0.014**	**−0.071**	**−0.016**	**0.002**	**0.040**	**0.957**^**a**^
		Egger	7	−0.115	0.067	−0.246	0.016	0.145	0.812	0.891^a^
		Weighted median	7	−0.039	0.023	−0.084	0.006	0.089	0.584	0.962^a^
		Weighted mode	7	−0.065	0.036	−0.137	0.006	0.123	0.913	0.937^a^
Schizophrenia	Accelerometer-based sedentary behaviour (G1)	IVW	3	**−0.230**	**0.028**	**−0.285**	**−0.175**	**0.000**	**0.000**	**0.795**^a^
		Egger	3	−0.079	0.893	−1.829	1.671	0.944	0.984	0.924^a^
		Weighted median	3	−0.217	0.198	−0.605	0.171	0.273	0.744	0.805^a^
		Weighted mode	3	−0.202	0.222	−0.636	0.232	0.458	0.913	0.817^a^
Schizophrenia	Accelerometer-based sedentary behaviour (G2)	IVW	53	**−0.260**	**0.087**	**−0.431**	**−0.089**	**0.003**	**0.045**	**0.771**^a^
		Egger	53	−0.362	0.268	−0.887	0.162	0.182	0.812	0.696^a^
		Weighted median	53	−0.207	0.079	−0.362	−0.052	0.009	0.186	0.813^a^
		Weighted mode	53	−0.227	0.137	−0.496	0.042	0.104	0.913	0.797^a^
		RAPS	53	**−0.259**	**0.047**	**−0.351**	**−0.166**	**0.000**	**0.000**	**0.772**^a^
Schizophrenia	Accelerometer-based walking (G1)	Wald ratio	1	**−0.998**	**0.322**	**−1.629**	**−0.368**	**0.002**	**0.040**	**0.368**^a^
Anorexia	Accelerometer-based sedentary behaviour (G2)	IVW	51	**−0.341**	**0.096**	**−0.530**	**−0.152**	**0.000**	**0.014**	**0.711**^a^
		Egger	51	−0.797	0.360	−1.503	−0.090	0.032	0.555	0.451^a^
		Weighted median	51	−0.278	0.136	−0.546	−0.011	0.041	0.351	0.757^a^
		Weighted mode	51	−0.287	0.281	−0.838	0.264	0.313	0.913	0.751^a^
		RAPS	52	**−0.280**	**0.086**	**−0.448**	**−0.111**	**0.001**	**0.012**	**0.756**^a^
ADHD	Self-reported moderate-vigorous PA (G2)	IVW	100	**0.525**	**0.171**	**0.189**	**0.860**	**0.002**	**0.040**	**1.690**^a^
		Egger	100	−0.517	0.716	−1.920	0.885	0.471	0.812	0.596^a^
		Weighted median	100	0.243	0.188	−0.125	0.611	0.195	0.744	1.275^a^
		Weighted mode	100	0.152	0.478	−0.786	1.090	0.751	0.913	1.164^a^
		RAPS	100	**0.545**	**0.120**	**0.310**	**0.780**	**0.000**	**0.000**	**1.725**^a^
Cigarette Smoking	Accelerometer-based PA, mean acceleration (G1)	IVW	6	**−0.026**	**0.005**	**−0.035**	**−0.017**	**0.000**	**0.000**	**−0.022**^b^
		Egger	6	−0.039	0.030	−0.097	0.019	0.261	0.812	−0.005^b^
		Weighted median	6	−0.022	0.009	−0.040	−0.004	0.018	0.276	−0.010^b^
		Weighted mode	6	−0.021	0.012	−0.044	0.003	0.145	0.913	−0.007^b^

Direction 2: Mental health/substance use disorders -> Physical activity/sedentary behaviour										
Exposure	Outcome	MR method	N (snps)	b	SE	CI (lower)	CI (upper)	*P*-value (raw)	*P*-value (FDR-adjusted)	Standardised effect
PTSD	Self-reported moderate-vigorous PA (G2)	IVW	220	**0.022**	**0.006**	**0.009**	**0.034**	**0.001**	**0.009**	**0.005**
		Egger	220	−0.012	0.027	−0.065	0.041	0.659	0.998	−0.001
		Weighted median	220	**0.025**	**0.007**	**0.011**	**0.039**	**0.001**	**0.020**	**0.005**
		Weighted mode	220	0.048	0.026	−0.002	0.098	0.063	0.872	0.003
		RAPS	220	**0.024**	**0.005**	**0.015**	**0.033**	**0.000**	**0.000**	**0.007**
Bipolar disorder	Accelerometer-based moderate PA (G1)	IVW	52	**0.043**	**0.013**	**0.017**	**0.068**	**0.001**	**0.010**	**0.016**
		Egger	52	0.139	0.063	0.015	0.263	0.033	0.796	0.011
		Weighted median	52	0.038	0.016	0.006	0.070	0.021	0.236	0.011
		Weighted mode	52	0.048	0.037	−0.024	0.120	0.198	0.872	0.006
Bipolar disorder	Accelerometer-based moderate PA (G2)	IVW	**234**	**0.024**	**0.007**	**0.011**	**0.037**	**0.000**	**0.006**	**0.018**
		Egger	234	0.022	0.023	−0.024	0.068	0.345	0.998	0.005
		Weighted median	234	0.027	0.009	0.008	0.045	0.005	0.082	0.014
		Weighted mode	234	0.042	0.028	−0.012	0.096	0.133	0.872	0.007
		RAPS	234	**0.027**	**0.006**	**0.015**	**0.040**	**0.000**	**0.000**	**0.021**
Bipolar disorder	Accelerometer-based sedentary behaviour (G2)	IVW	234	**−0.026**	**0.008**	**−0.041**	**−0.011**	**0.001**	**0.009**	**−0.017**
		Egger	234	−0.016	0.027	−0.070	0.038	0.565	0.998	−0.003
		Weighted median	234	−0.013	0.010	−0.032	0.006	0.176	0.742	−0.007
		Weighted mode	234	0.014	0.029	−0.042	0.070	0.619	0.872	0.002
		RAPS	234	**−0.027**	**0.006**	**−0.039**	**−0.014**	**0.000**	**0.000**	**−0.021**
Schizophrenia	Accelerometer-based moderate PA (G2)	IVW	424	**0.018**	**0.003**	**0.011**	**0.024**	**0.000**	**0.044**	**0.014**
		Egger	424	0.024	0.012	0.002	0.047	0.035	0.998	0.004
		Weighted median	424	0.013	0.004	0.006	0.021	0.001	0.215	0.012
		Weighted mode	424	0.009	0.013	−0.017	0.035	0.511	0.872	0.006
		RAPS	424	**0.018**	**0.002**	**0.014**	**0.023**	**0.000**	**0.021**	**0.014**
Schizophrenia	Accelerometer-based sedentary behaviour (G2)	IVW	424	**−0.023**	**0.006**	**−0.035**	**−0.011**	**0.000**	**0.006**	**−0.018**
		Egger	424	−0.023	0.022	−0.066	0.021	0.309	0.998	−0.005
		Weighted median	424	0.002	0.008	−0.013	0.018	0.777	0.944	0.001
		Weighted mode	424	0.018	0.021	−0.023	0.060	0.388	0.872	0.004
		RAPS	424	**−0.022**	**0.005**	**−0.031**	**−0.012**	**0.000**	**0.000**	**−0.021**
Schizophrenia	Self-reported moderate-vigorous PA (G1)	IVW	196	**0.017**	**0.005**	**0.008**	**0.026**	**0.000**	**0.004**	**0.009**
		Egger	196	0.021	0.018	−0.015	0.057	0.255	0.998	0.003
		Weighted median	196	0.009	0.005	−0.001	0.018	0.076	0.685	0.004
		Weighted mode	196	−0.002	0.013	−0.027	0.024	0.883	0.935	−0.000
Schizophrenia	Self-reported moderate-vigorous PA (G2)	IVW	**424**	**0.018**	**0.003**	**0.011**	**0.024**	**0.000**	**0.000**	**0.013**
		Egger	424	0.024	0.012	0.002	0.047	0.035	0.796	0.005
		Weighted median	424	**0.013**	**0.004**	**0.006**	**0.021**	**0.001**	**0.020**	**0.008**
		Weighted mode	424	0.009	0.013	−0.017	0.035	0.511	0.872	0.002
		RAPS	424	**0.018**	**0.002**	**0.014**	**0.023**	**0.000**	**0.000**	**0.018**
Anorexia	Self-reported moderate-vigorous PA (G1)	IVW	6	**0.061**	**0.009**	**0.042**	**0.079**	**0.000**	**0.000**	**0.015**
		Egger	6	0.051	0.037	−0.021	0.124	0.238	0.998	0.003
		Weighted median	6	**0.065**	**0.015**	**0.035**	**0.094**	**0.000**	**0.001**	**0.010**
		Weighted mode	6	0.071	0.022	0.029	0.114	0.021	0.872	0.008
ADHD	Self-reported moderate-vigorous PA (G2)	IVW	73	**0.017**	**0.005**	**0.007**	**0.027**	**0.001**	**0.009**	**0.008**
		Egger	73	0.015	0.018	−0.020	0.049	0.405	0.998	0.002
		Weighted median	73	0.008	0.006	−0.004	0.020	0.191	0.742	0.003
		Weighted mode	73	−0.006	0.015	−0.036	0.024	0.691	0.899	−0.001
		RAPS	73	**0.013**	**0.004**	**0.006**	**0.020**	**0.000**	**0.003**	**0.009**
Autism	Accelerometer-based walking (G1)	IVW	2	**−0.122**	**0.021**	**−0.164**	**−0.081**	**0.000**	**0.000**	**−0.027**

Only IVW/Wald ratio estimates with a FDR-adjusted *p* < 0.05 are presented in the table (the full MR results for all associations are reported in Appendix 2); alternative MR methods are not available for instruments including less than 3 SNPs; as effect size measures, odds ratios are reported for binary outcomes and standardised beta coefficients for continuous outcomes;

*MR* Mendelian randomisation, *IVW* inverse variance weighted, *RAPS* Robust adjusted profile score, *CI* confidence interval, *FDR* false discovery rate, *PA* physical activity;

G1 = genome-wide significant genetic instrument (*P* < 5 × 10^ − 8^), G2 = more relaxed genetic instrument (*P* < 1 × 10^ − 6^).

^a^Odds ratio.

^b^standardised effect size.

Bold values indicate MR effects with a *p*-value < 0.05.

### Main analyses

#### Direction 1: Association of genetically predicted physical activity/sedentary behaviour with mental health/substance use disorders

Higher levels of genetically predicted accelerometer-based average physical activity had a protective association with depression (G1: IVW b = −0.043, 95% CI: −0.071 to −0.016) and cigarette smoking (G1: IVW b = −0.026, 95% CI: −0.035 to −0.017). Genetically predicted accelerometer-based sedentary behaviour was associated with a lower risk of schizophrenia at both instrument thresholds (G1: IVW b = −0.230, 95% CI: −0.285 to −0.175; G2: IVW b = −0.260, 95% CI: −0.431 to −0.089), and genetically predicted accelerometer-based walking had a protective association with schizophrenia (G1: WR b = −0.998, 95% CI: −1.629 to −0.368). However, the latter association was driven by a single SNP, and its direction was inconsistent when using the more relaxed instrument threshold. Genetically predicted sedentary behaviour also had a protective association with anorexia nervosa (G2: IVW b = −0.341, 95% CI: −0.530 to −0.152). Genetically predicted self-reported moderate-to-vigorous activity was associated with a higher risk of ADHD (G2: IVW b = 0.525, 95% CI: 0.189 to 0.860) (Fig. 2). The odds ratios of these associations ranged from small to moderate [43] (Table 2). As expected, genetically predicted physical activity was not associated with birth length (i.e., negative control outcome; eTable 3).

#### Direction 2: Association of genetically predicted mental health/substance use disorders with physical activity/sedentary behaviour

Genetically predicted PTSD was associated with higher levels of self-reported physical activity (G2: IVW b = 0.022, 95% CI: 0.009 to 0.034). Genetically predicted bipolar disorder was associated with lower levels of sedentary behaviour (G2: IVW b = −0.026, 95% CI: −0.041 to −0.011), and we also observed a positive association between genetically predicted bipolar disorder and accelerometer-based moderate activity at both instrument thresholds (G1: IVW b = 0.043, 95% CI: 0.017 to 0.068, *p* = 0.001; G2: IVW b = 0.024, 95% CI: 0.011 to 0.037, *p* < 0.001). Genetically predicted schizophrenia was associated with lower levels of accelerometer-based sedentary behaviour (G2: IVW b = −0.023, 95% CI: −0.035 to −0.011), with higher levels of accelerometer-based moderate activity (G2: IVW b = 0.018, 95% CI: 0.011 to 0.024), and with higher levels of self-reported moderate-to-vigorous physical activity (G1: IVW b = 0.017, 95% CI: 0.008 to 0.026; G2: IVW b = 0.018, 95% CI: 0.011 to 0.024). Genetically predicted anorexia (G1: IVW b = 0.061, 95% CI: 0.042 to 0.079) and ADHD (G2: IVW b = 0.017, 95% CI: 0.007 to 0.027) were associated with higher levels of self-reported physical activity. Genetically predicted autism was associated with reduced levels of accelerometer-based walking (G1: IVW b = −0.122, 95% CI: −0.164 to −0.081) (Fig. 3). However, the effect size of these associations was generally small (Table 2).

### Sensitivity analyses

The results of the sensitivity analyses with MR-Egger, weighted median, and weighted mode revealed associations in the same direction as those observed in the main analyses, but the confidence intervals were often imprecise (Table 2). Of note, these sensitivity methods have lower statistical power than IVW because they rely on stricter assumptions, and therefore their results are expected to provide weaker statistical evidence but not effect sizes. MR-RAPS provided consistent and precise results across most outcomes (Table 2). The intercept of MR-Egger (eTable 8), Q statistics (eTable 7), and MR-PRESSO (eTable 10) provided little evidence of heterogeneity and unbalanced horizontal pleiotropy in the association of genetically predicted physical activity/sedentary behaviour with depression, anorexia, and cigarette smoking and in the association of genetically predicted anorexia with physical activity. In contrast, Q statistics and MR-PRESSO tests highlighted the presence of heterogeneous associations and outliers in the G2 instrument relationship of genetically predicted sedentary behaviour and self-reported physical activity with depression and ADHD, respectively, and in the associations of genetically predicted PTSD bipolar disorder, schizophrenia, and ADHD with physical activity/sedentary behaviour. These associations were generally smaller and more precise following the removal of outliers by MR-PRESSO. The Steiger test for the average association of all the genetic variants associated with a particular phenotype suggested that the overall direction of the observed MR associations was correct. When considering the associations of individual SNPs, we found evidence of misspecified SNPs in the MR analysis of self-reported physical activity and ADHD. Their association was considerably smaller and no longer consistent with one direction after their removal by Steiger filtering, thereby suggesting that self-reported physical activity was not precisely associated with ADHD. We also observed misspecified SNPs in the genetic instruments for bipolar disorder and schizophrenia, but the magnitude and precision of their relationship with physical activity did not change substantially after applying Steiger filtering (eTable 11).

## Discussion

Using data from large-scale GWASs, we applied two-sample MR to test whether physical activity and sedentary behaviour are causally associated with mental health and substance use disorders, or vice versa. The results showed that objectively assessed but not self-reported physical activity had a protective association with depression and cigarette smoking. In contrast, objectively assessed sedentary behaviour had a protective association with anorexia and schizophrenia, and objectively assessed walking was associated with a lower risk of schizophrenia. We also found evidence of a causal association between mental health disorders and physical activity. Specifically, PTSD, schizophrenia, anorexia, and ADHD were all associated with higher levels of self-reported physical activity. Furthermore, schizophrenia and bipolar disorder were associated with higher levels of objectively assessed moderate activity and with reduced levels of sedentary behaviour, whereas autism was associated with lower walking activity. These findings highlight the important but complex nature in which physical activity and sedentary behaviour are related to mental health and substance use disorders.

### Causal pathways between physical activity and depression and cigarette smoking

Earlier results from RCTs and prospective cohort studies suggest that physical activity, measured through either self-report or objective methods, can reduce the risk of depression across the population and ameliorate depressive symptoms not only among depressed patients, but also in patients with other mental and physical health conditions [16, 44]. Furthermore, a previous MR study found evidence of a causal protective relationship between objective but not self-reported physical activity and depression, which was not observed in the opposite direction [26]. Correspondingly, the results presented here indicate a 5% reduction in the odds of depression for every 1 standard deviation (SD) increase in objectively assessed average physical activity. Our results also extend earlier MR findings by showing that other intensity levels of physical activity (i.e., moderate activity and walking) and sedentary behaviour were not associated with depression. Furthermore, in the opposite direction of causation, depression showed weak associations with all physical activity outcomes assessed in this study. Taken together, these results suggest that increasing overall levels of physical activity may be an effective strategy to prevent and treat depression.

Exercise has been proposed as an additional treatment for smoking cessation because it can help to relieve nicotine withdrawal symptoms and smoking craving. However, RCTs have provided mixed findings regarding the efficacy of physical activity interventions for smoking cessation. Accordingly, a meta-analysis of RCTs did not find consistent evidence of an effect of different types of physical activities (e.g., aerobic exercise, yoga) on smoking cessation [45]. There also is limited evidence regarding the protective effects of physical activity on smoking initiation or the levels of smoking among current smokers, although initial findings from prospective cohort studies indicate that physical activity is prospectively associated with a reduced risk of smoking [46]. Our results suggest that every 1 SD increase in objectively assessed average activity may result in 0.26 fewer cigarettes smoked per day. Other types/intensity levels of physical activity and sedentary behaviour were not associated with the risk of smoking. In the opposite direction of causation, we also found weak evidence of a causal association between cigarette smoking and physical activity/sedentary behaviour. These results corroborate earlier findings from observational studies, suggesting that enhancing physical activity levels could be an effective strategy to reduce the risk of smoking across the general population.

### Causal pathways between physical activity and schizophrenia, PTSD, and bipolar disorder

RCTs suggest that high-intensity physical activity interventions and aerobic exercise can improve psychiatric symptoms, cognitive function, and quality of life in patients with schizophrenia, PTSD, and bipolar disorder [47, 48]. Observational studies further suggest that physical activity could reduce the risk of these disorders in the population [12], but this relationship is small when accounting for confounding factors, and most studies that have been conducted to date are cross-sectional [12, 49]. Furthermore, an earlier MR study found that physical activity was a protective factor for bipolar disorder but not for schizophrenia [27]. Our results indicate that a 1 SD increase in the amount of sedentary behaviour can reduce the odds of schizophrenia by 20%. We also observed a 60% reduction in the risk of schizophrenia for every 1 SD increase in walking activity. In the opposite direction of causation, schizophrenia was associated with lower levels of sedentary behaviour, as well as being associated with higher levels of objectively assessed moderate activity and self-reported physical activity, suggesting that reverse causality might be at play. Furthermore, we found weak evidence for the plausible protective association of physical activity with bipolar disorder and PTSD. The result for bipolar disorder contradicts earlier MR evidence suggesting a protective association between physical activity and bipolar disorder [27]. Such discrepancy could be explained by the use of a newer and larger GWAS dataset for bipolar disorder in our study. In the opposite direction of causation, both PTSD and bipolar disorder were associated with increased levels of physical activity. Increased physical activity might, thus, reflect psychopathological symptoms, such as high energy levels and disorganisation in mania or engagement in demanding activities to avoid re-experiencing in PTSD. These results outline the complex nature of the links of physical activity/sedentary behaviour with schizophrenia, bipolar disorder, and PTSD, and they suggest that increasing levels of physical activity might not be an effective strategy to reduce the risk of these disorders. Further research is needed to better understand the impact of different types and intensity levels of physical activity for the prevention and treatment of these disorders.

### Causal pathways between physical activity and eating disorders

Observational studies suggest that people with eating disorders often engage in excessively high levels of physical activity and have hyperactive lifestyles in order to maximise energy expenditure and weight loss, either as a conscious strategy or because of a subconscious biological drive [50]. Our results partly align with earlier findings, as they show that anorexia is associated with higher levels of self-reported physical activity. However, the association between anorexia and objective physical activity was weak. This could indicate that this disorder may have a greater impact on the subjective experience of physical activity than on the actual levels of physical activity undertaken. This result should be further explored in observational studies comparing the association of anorexia with self-reported versus objective physical activity levels. Another novel result is that the odds of anorexia decreased by 30% for every 1 SD increase in the levels of sedentary behaviour. This result is consistent with the current clinical guidelines for the treatment of anorexia, which recommend stopping vigorous exercise to facilitate recovery [51]. Therefore, enhancing sedentary behaviours and light activities involving minimal energy expenditure could be an effective strategy to prevent and treat the physical and psychological symptoms of anorexia and other eating disorders.

### Causal pathways between physical activity and neurodevelopmental disorders

Initial evidence from clinical trials suggests that interventions involving physical activity might help to ameliorate certain symptoms of neurodevelopmental disorders including ADHD and autism. However, only a paucity of studies have tested this relationship, and the quality of the available evidence is weak [52–54]. Our results provide weak evidence of a protective association between physical activity and ADHD and autism. In the opposite direction of causation, ADHD was associated with higher levels of self-reported physical activity, whereas autism was associated with reduced levels of objectively assessed walking. Of note, these results are consistent with the findings of a recent UK Biobank study showing that genetic liability to ADHD is associated with higher levels of physical activity, while genetic liability to autism is linked to reduced physical activity [55]. However, it is worth noting that the GWASs of ADHD and autism were largely conducted in children and young people, whereas the GWAS of physical activity used in this study was based on a sample of adults. These findings could therefore be inconclusive if the genetic determinants of physical activity in childhood are different from those in adulthood.

### Strengths and limitations

Our study has several strengths, including (i) the application of a genetically informed approach to strengthen causal inferences; (ii) the use of summary statistics drawn from the largest available GWASs with non-overlapping samples for each exposure-outcome relationship; (iii) the inclusion of different types of physical activity phenotypes based on both self-reported and objectively assessed data; (iv) a comprehensive assessment of the role of physical activity across a variety of mental health and substance use disorders; (v) the use of several sensitivity analyses and robust MR methods to ascertain the validity of key MR assumptions and assess the accuracy of the results; (vi) and the inclusion of a negative control outcome that, as expected, was not causally affected by any physical activity exposure included in our analysis.

Despite these strengths, the results should be interpreted in light of some limitations [56]. First, associations from MR do not provide information on temporal patterns and should be interpreted as the lifetime effects of the liability to a particular risk factor. In addition, our measures of mental health/substance use disorders represent prevalent cases, so our results cannot clearly disentangle the role of physical activity in the prevention versus treatment of mental illness. Second, some of the included GWASs only identified few genome-wide significant SNPs associated with the exposures of interest (e.g., objectively assessed physical activity; see eTable 1, Appendix 1), which could affect the power of the instruments. To address this, we used a second set of genetic instruments including top SNPs meeting a more relaxed *p*-value threshold, and we applied MR-RAPS to account for weak instrument bias. In addition, although we used the largest available GWASs, some were based on relatively small samples. Hence, the weak associations between physical activity and certain outcomes (e.g., autism, alcohol dependence) observed in our study could be explained by methodological issues related to the power of the instruments and the GWAS datasets, which might have increased the risk of false negative results (i.e., Type 2 error). Moreover, common SNPs usually explain a limited proportion of the total variance in complex traits, and their exact biological action is unclear to date. As such, we cannot rule out the possibility that pleiotropic mechanisms might have affected the main study results. Third, it should be noted that genetic variants linked to physical activity are correlated with a variety of cognitive and physical traits, such as intelligence, body composition, and metabolic factors [29], which are all associated with mental health. Notably, these traits could represent alternative pathways through which genetic variants linked to physical activity may affect mental health and could therefore be possible sources of horizontal pleiotropy. Future research could further explore the role of pleiotropic effects using multivariable MR to test the direct effects of physical activity on mental health and substance use disorders after controlling for potential confounding factors (e.g., intelligence, educational attainment, body mass index), as well as novel MR approaches such as PheWAS-based clustering of Mendelian Randomisation instruments [57]. Fourth, we found evidence of a bidirectional causal relationship between sedentary behaviour and schizophrenia. However, a causal effect in both directions could be a product of violations of the second and third IV assumptions (see Fig. 1) (e.g., the genetics of personality or intelligence may influence both physical activity and mental illness) rather than indicating a true bidirectional relationship [25]. Lastly, the genetic instruments for physical activity were all identified in the UK Biobank, which only includes adults aged 40 to 70 years and is not representative of the wider UK population. Furthermore, we do not have detailed information on the demographic characteristics of the participants included in the GWASs of mental health and substance use disorders. Therefore, our findings might not be generalisable to other populations and might have been affected by participation bias, which could influence both the strength and direction of the links between physical activity and mental health.

### Clinical implications

Physical activity may be an effective strategy to reduce the risk of depression and cigarette smoking across the population and treat these disorders amongst those affected. Of note, physical activity interventions have been shown to reduce depressive symptoms in individuals affected by other mental disorders (e.g., schizophrenia, PTSD, anxiety, autism), as well as improving physical health and cognitive function [16, 44]. While the benefits of exercise for both mental and physical health are generally well recognised, physical activity is often overlooked in prevention and treatment programmes for mental health and substance use disorders, and physical activity interventions are not routinely available as a treatment option for patients. An important issue to consider is that psychiatric disorders are a complex and highly heterogenous group of disorders, which are characterised by a multitude of symptoms, risk factors, and consequences, and this may affect the efficacy and effectiveness of physical activity interventions across different disorders. Accordingly, our results highlight the complex links between physical activity and psychiatric disorders and suggest that physical activity may be effective for specific types of symptoms, including depressive symptoms and addictive behaviours. Furthermore, more research is needed to clearly disentangle the effects of specific types and intensity levels of physical activity on different mental health and substance use disorders. For instance, a rapidly growing body of research indicates that body-mind activities (e.g., yoga) and low intensity activities (e.g., walking) have positive effects on various mental health disorders [58–60]. Correspondingly, the results presented here suggest that sedentary and light physical activities could be particularly beneficial for certain disorders, such as anorexia and schizophrenia. As such, a systematic assessment of the role of different types and intensity levels of physical activity both within and between psychiatric disorders is warranted in future studies.

## Conclusions

In summary, this study capitalises on a genetically informed approach to test the plausible protective effects of physical activity on ten psychiatric disorders. Our results suggest that physical activity has a protective association with depression (in line with earlier MR evidence) and cigarette smoking, whereas sedentary behaviour is associated with a reduced risk of anorexia and schizophrenia. Furthermore, they outline the likely impact of mental illness on physical activity levels, and they also point to the importance of considering different assessment methods, types, and intensity levels of physical activity in mental health research. Programmes to enhance physical activity may be an effective strategy to reduce the risk of depression and cigarette smoking. In contrast, the promotion of sedentary or light physical activities could help to reduce the risk of anorexia nervosa and other severe mental disorders.

### Supplementary information

Appendix 1

Appendix 2

## Data Availability

Summary-level data for the exposures and outcomes were drawn from large-scale GWASs or genetic consortia, including the UK Biobank, the Psychiatric Genomics Consortium, the GWAS and Sequencing Consortium of Alcohol and Nicotine Use, and the Early Growth Consortium.

## Code availability

The code of the statistical analyses can be accessed on GitHub: https://github.com/Ellie25moon/2-Sample-MR-study-of-physical-activity-and-mental-health-.
