# Supplementary material for: Testing the causal relationships of physical activity and sedentary behaviour with mental health and substance use disorders: a Mendelian randomisation study
Source: Mol Psychiatry. 2023 Jul 21;28(8):3429–43. doi: 10.1038/s41380-023-02133-9 (PMC10618087; doi:10.1038/s41380-023-02133-9)
Supplement: Supplementary file 1 — Appendix 1 [file 41380_2023_2133_MOESM1_ESM.docx]

**APPENDIX 1**

**Testing the causal relationships of physical activity and sedentary behaviour with mental health and substance use disorders: A Mendelian Randomisation study.**

Eleonora Iob, Jean-Baptiste Pingault, Marcus R. Munafò, Brendon Stubbs, Mark S. Gilthorpe, Adam X. Maihofer, Psychiatric Genomics Consortium Posttraumatic Stress Disorder Working Group, Andrea Danese

**Contents**

**eMETHODS**

- eTable1. Overview of the GWAS datasets.
- Deviations from the pre-registered study protocol.

**SUPPLEMENTARY FIGURES**

- eFigure1. MR estimates and 95% confidence intervals for the causal relationships of physical activity and sedentary behaviour with mental health and substance use disorders.
- eFigure2. MR estimates and 95% confidence intervals for the causal relationships of mental health and substance use disorders with physical activity and sedentary behaviour.

**eMETHODS**

| eTable1. Overview of the GWAS datasets. | | | | | | |
| --- | --- | --- | --- | --- | --- | --- |
| Phenotype | **Consortium** | **Total sample size** | **SNP-based heritability** | **Z-value** | **Genome-wide significant loci** | **Source** |
| *a) Physical activity and sedentary behaviour (exposure)* | | |  |  |  |  |
| Self-reported moderate-to-vigorous physical activity | UK Biobank | ﻿377,234 | 0.05 | 21.74 | 9 | ^1^ |
| Accelerometer-based physical activity (average acceleration) | UK Biobank | ﻿ ﻿91,105 | 0.14 | 16.87 | 2 | ^1^ |
| Accelerometer-based moderate activity | UK Biobank | 91,105 | 0.10 | 33.33 | 1 | ^2^ |
| Accelerometer-based walking | UK Biobank | 91,105 | 0.12 | 40.00 | 1 | ^2^ |
| Accelerometer-based sedentary behaviour | UK Biobank | 91,105 | 0.15 | 50.00 | 6 | ^2^ |
| *b) Mental health disorders (outcome)* | |  |  |  |  |  |
| Major depressive disorder | PGC | ﻿143 265 | 0.09 | 22.50 | 44 | ^3^ |
| Post-traumatic stress disorder (PTSD) | PGC | 956,337 | 0.06 | 26.09 | 46 | Unpublished |
| Bipolar disorder | PGC | 413,466 | 0.19 | 23.75 | 64 | ^4^ |
| Schizophrenia | PGC | 306,011 | 0.24 | 24.00 | 287 | ^5^ |
| Anorexia nervosa | PGC | 68,684 | 0.11­­-0.17 | 11.00-17.00 | 8 | ^6^ |
| ﻿Attention deficit/hyperactivity disorder (ADHD) | ﻿PGC | 55,374 | 0.22 | 15.71 | 12 | ^7^ |
| Autism spectrum disorder | PGC | 46,350 | 0.12 | 12.00 | 5 | ^8^ |
| *c) Substance use disorders (outcome)* | |  |  |  |  |  |
| Alcohol dependence | PGC | ﻿46,568 | 0.09 | 4.74 | 1 | ^9^ |
| Cannabis use disorder | PGC | 374,287 | ﻿0.07-0.12 | 11.67 | 2 | ^10^ |
| Cigarette smoking  (number of cigarettes smoked per day) | ﻿GWAS and Sequencing Consortium of Alcohol and Nicotine use | 143,210 | 0.08 | 10.00 | 72 | ^11^ |
| *d) Negative control outcome* | |  |  |  |  |  |
| Infant birth length | EGC | 28,459 | 0.05 | 6.25 | 7 | ^12^ |
| *Note. PGC = Psychiatric Genomics Consortium; EGC = Early Growth Consortium. The power of a GWAS dataset should be sufficient if there is at least one genome-wide significant locus, the SNP-based heritability is ≥ 0.05, and the Z-value is ≥ 4.*^13^ | | | | | | |

**Deviations from the pre-registered study protocol**

Our original pre-registered study protocol also included anxiety disorders, obsessive compulsive disorder (OCD), and opioid dependence as primary outcomes/exposures. However, we have decided to exclude these disorders as their GWAS datasets may have limited power, as shown below.

| Phenotype | Consortium | Total sample size | SNP-based heritability | Z-value | Genome-wide significant loci | Source |
| --- | --- | --- | --- | --- | --- | --- |
| Anxiety disorders | PGC | ﻿17,310 | ﻿0.10 | 2.50 | 1 | ^14^ |
| Obsessive compulsive disorder (OCD) | ﻿PGC | 9,725 | 0.28 | 7.00 | 0 | ^15^ |
| Opioid dependence (vs ﻿opioid-unexposed controls) | PGC | 28,709 | 0.28 | 2.80 | 0 | ^16^ |
| *Note. The power of a GWAS dataset should be sufficient if there is at least one genome-wide significant locus, the SNP-based heritability is ≥ 0.05, and the Z-value is ≥ 4.*^13^ | | | | | | |

**
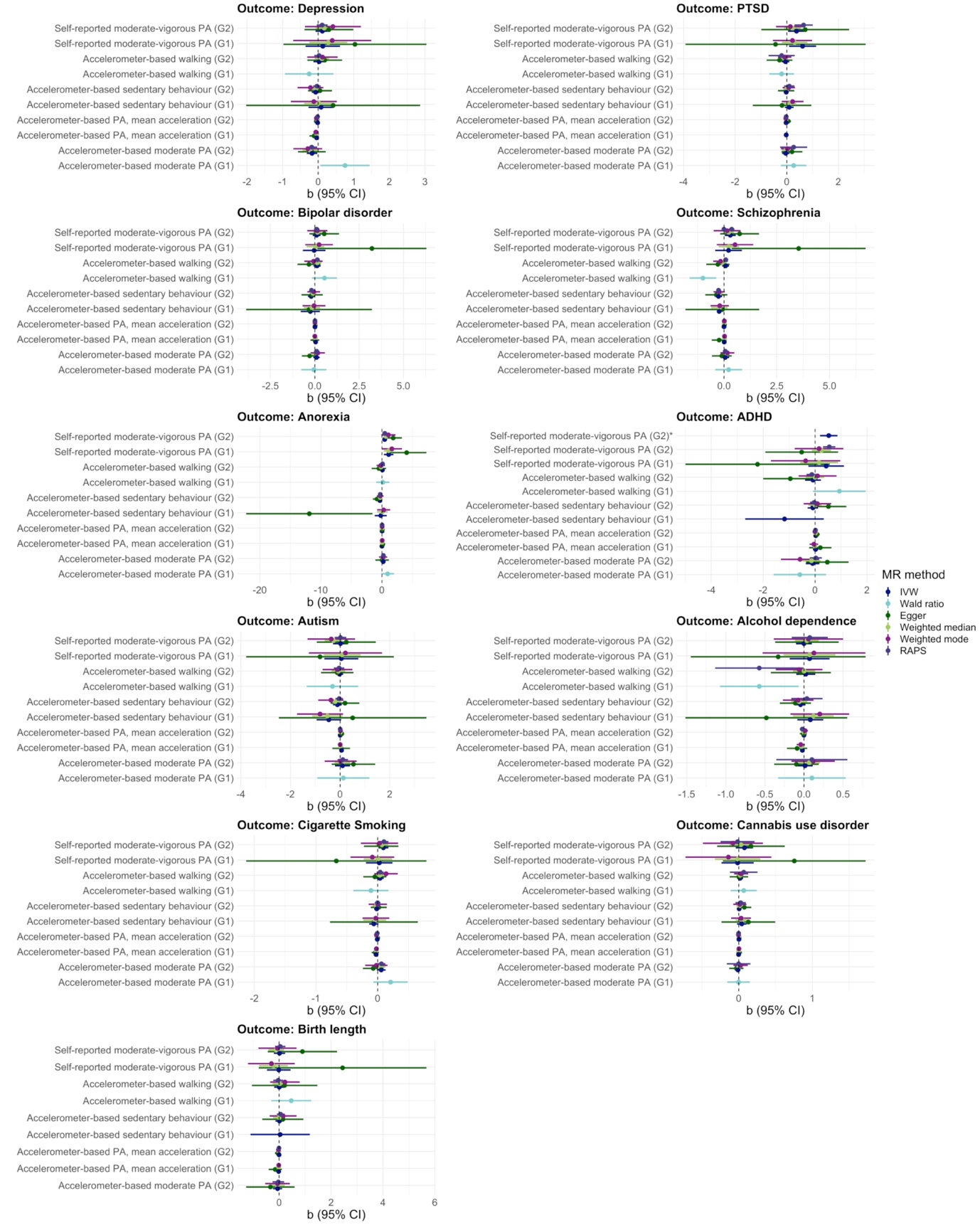
**

**eFigure1. MR estimates and 95% confidence intervals for the causal relationships of physical activity and sedentary behaviour with mental health and substance use disorders.**

**Note.** MR = Mendelian randomisation; IVW = inverse variance weighted; RAPS = Robust adjusted profile score; CI = confidence interval; G1 = genome-wide significant genetic instrument (P < 5×10^−8^); G2 = more relaxed genetic instrument (P < 1×10^−6^); PA = physical activity.

**
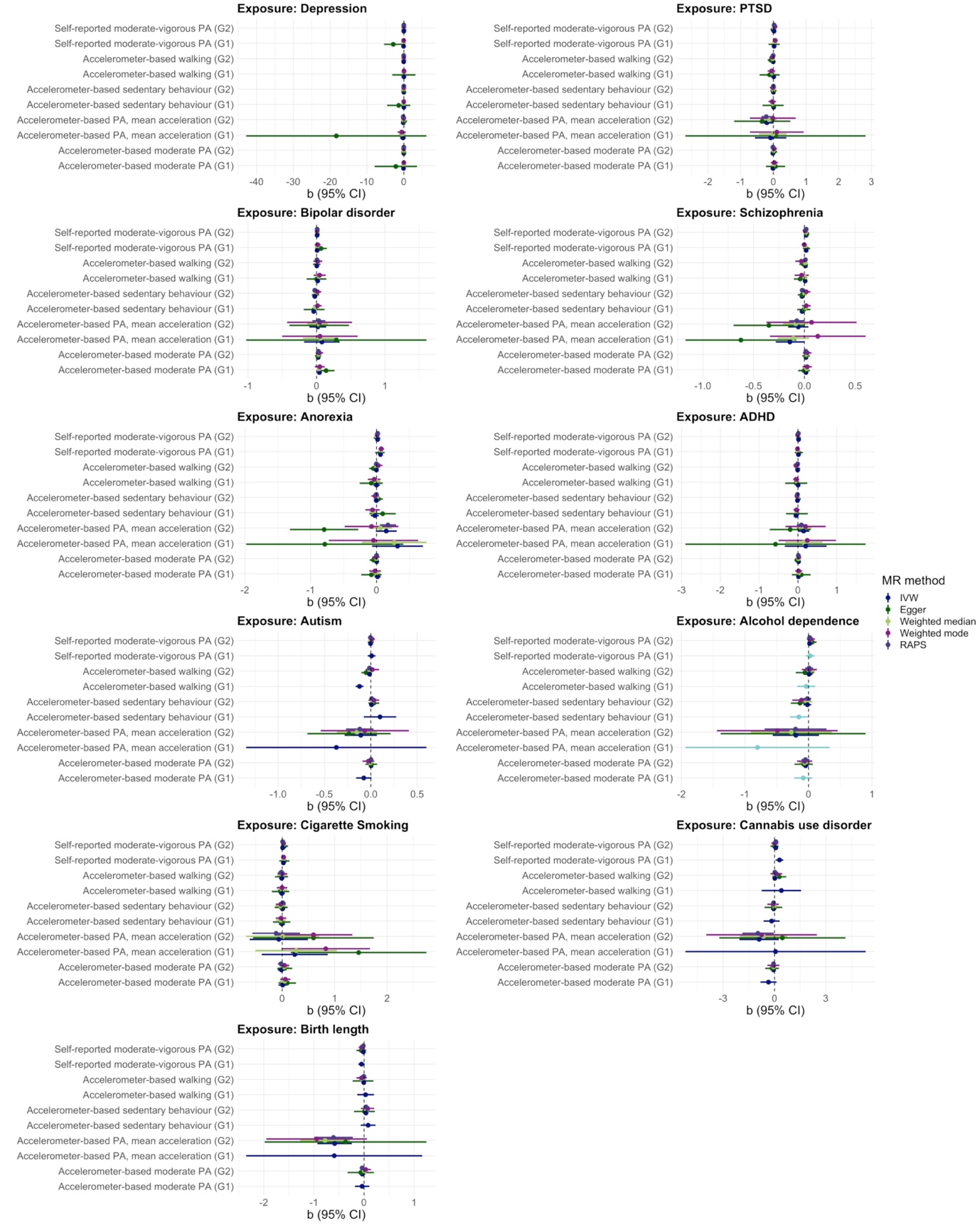
**

**eFigure2. MR estimates and 95% confidence intervals for the causal relationships of mental health and substance use disorders with physical activity and sedentary behaviour.**

**Note.** MR = Mendelian randomisation; IVW = inverse variance weighted; RAPS = Robust adjusted profile score; CI = confidence interval; G1 = genome-wide significant genetic instrument (P < 5×10^−8^); G2 = more relaxed genetic instrument (P < 1×10^−6^); PA = physical activity.

**REFERENCES**

1 Klimentidis YC, Raichlen DA, Bea J, Garcia DO, Wineinger NE, Mandarino LJ *et al.* Genome-wide association study of habitual physical activity in over 377,000 UK Biobank participants identifies multiple variants including CADM2 and APOE. *Int J Obes* 2018; **42**: 1161–1176.

2 Doherty A, Smith-Byrne K, Ferreira T, Holmes M V., Holmes C, Pulit SL *et al.* GWAS identifies 14 loci for device-measured physical activity and sleep duration. *Nat Commun 2018 91* 2018; **9**: 1–8.

3 Wray NR, Ripke S, Mattheisen M, Trzaskowski M, Byrne EM, Abdellaoui A *et al.* Genome-wide association analyses identify 44 risk variants and refine the genetic architecture of major depression. *Nat Genet* 2018; **50**: 668–681.

4 Mullins N, Forstner AJ, O’Connell KS, Coombes B, Coleman JRI, Qiao Z *et al.* Genome-wide association study of more than 40,000 bipolar disorder cases provides new insights into the underlying biology. *Nat Genet* 2021; **53**: 817–829.

5 Trubetskoy V, Pardiñas AF, Qi T, Panagiotaropoulou G, Awasthi S, Bigdeli TB *et al.* Mapping genomic loci implicates genes and synaptic biology in schizophrenia. *Nature* 2022; **604**: 502–508.

6 Watson HJ, Yilmaz Z, Thornton LM, Hübel C, Coleman JRI, Gaspar HA *et al.* Genome-wide association study identifies eight risk loci and implicates metabo-psychiatric origins for anorexia nervosa. *Nat Genet 2019 518* 2019; **51**: 1207–1214.

7 Demontis D, Walters RK, Martin J, Mattheisen M, Als TD, Agerbo E *et al.* Discovery of the first genome-wide significant risk loci for attention deficit/hyperactivity disorder. *Nat Genet* 2019; **51**: 63–75.

8 Grove J, Ripke S, Als TD, Mattheisen M, Walters RK, Won H *et al.* Identification of common genetic risk variants for autism spectrum disorder. *Nat Genet* 2019; **51**: 431–444.

9 Walters RK, Polimanti R, Johnson EC, McClintick JN, Adams MJ, Adkins AE *et al.* Transancestral GWAS of alcohol dependence reveals common genetic underpinnings with psychiatric disorders. *Nat Neurosci* 2018; **21**: 1656–1669.

10 Johnson EC, Demontis D, Thorgeirsson TE, Walters RK, Polimanti R, Hatoum AS *et al.* A large-scale genome-wide association study meta-analysis of cannabis use disorder. *The Lancet Psychiatry* 2020; **7**: 1032–1045.

11 Liu M, Jiang Y, Wedow R, Li Y, Brazel DM, Chen F *et al.* Association studies of up to 1.2 million individuals yield new insights into the genetic etiology of tobacco and alcohol use. *Nat Genet 2019 512* 2019; **51**: 237–244.

12 van der Valk RJP, Kreiner-Møller E, Kooijman MN, Guxens M, Stergiakouli E, Sääf A *et al.* A novel common variant in DCST2 is associated with length in early life and height in adulthood. *Hum Mol Genet* 2015; **24**: 1155–1168.

13 Watanabe K, Stringer S, Frei O, Umićević Mirkov M, de Leeuw C, Polderman TJC *et al.* A global overview of pleiotropy and genetic architecture in complex traits. *Nat Genet* 2019; **51**: 1339–1348.

14 Otowa T, Hek K, Lee M, Byrne EM, Mirza SS, Nivard MG *et al.* Meta-analysis of genome-wide association studies of anxiety disorders. *Mol Psychiatry* 2016; **21**: 1391–1399.

15 IOCDF-GC and OCGAS. Revealing the complex genetic architecture of obsessive–compulsive disorder using meta-analysis. *Mol Psychiatry* 2018; **23**: 1181–1188.

16 Polimanti R, Walters RK, Johnson EC, McClintick JN, Adkins AE, Adkins DE *et al.* Leveraging genome-wide data to investigate differences between opioid use vs. opioid dependence in 41,176 individuals from the Psychiatric Genomics Consortium. *Mol Psychiatry* 2020; **25**: 1673–1687.
